# Differential contributions of body form, motion, and temporal information to subjective action understanding in naturalistic stimuli

**DOI:** 10.3389/fnint.2024.1302960

**Published:** 2024-03-12

**Authors:** Vojtěch Smekal, Marta Poyo Solanas, Evelyne I. C. Fraats, Beatrice de Gelder

**Affiliations:** Brain and Emotion Lab, Department of Cognitive Neuroscience, Maastricht Brain Imaging Centre, Maastricht University, Maastricht, Netherlands

**Keywords:** action perception, emotion, human body, naturalistic stimuli, subjective report

## Abstract

**Introduction:**

We investigated the factors underlying naturalistic action recognition and understanding, as well as the errors occurring during recognition failures.

**Methods:**

Participants saw full-light stimuli of ten different whole-body actions presented in three different conditions: as normal videos, as videos with the temporal order of the frames scrambled, and as single static representative frames. After each stimulus presentation participants completed one of two tasks—a forced choice task where they were given the ten potential action labels as options, or a free description task, where they could describe the action performed in each stimulus in their own words.

**Results:**

While generally, a combination of form, motion, and temporal information led to the highest action understanding, for some actions form information was sufficient and adding motion and temporal information did not increase recognition accuracy. We also analyzed errors in action recognition and found primarily two different types.

**Discussion:**

One type of error was on the semantic level, while the other consisted of reverting to the kinematic level of body part processing without any attribution of semantics. We elaborate on these results in the context of naturalistic action perception.

## 1 Introduction

Early psychological research investigating biological motion perception used point-light displays–lights affixed to the joints of the human body (Cutting and Kozlowski, 1977; Walk and Homan, 1984). Observing the motion of the points was enough for participants to perceive the form of a human body and decode its movements [Johansson (1973), see Blake and Shiffrar (2007) for a review of point-light display studies], as well as recognizing locomotory, instrumental, and social actions (Dittrich, 1993; Bertenthal and Pinto, 1994).

Studies investigating the neural basis of action perception have also taken advantage of point-light displays (Vaina et al., 2001; Grossman and Blake, 2002; Centelles et al., 2011), but there is an increasing demand in the neuroscientific community for more naturalistic stimuli and paradigms, which may better reflect processes that occur in day-to-day life (Sonkusare et al., 2019; de Gelder and Poyo Solanas, 2021; Miller et al., 2022). Additionally, as Troje (2008) notes, action recognition is only one of the processing levels that constitutes biological motion perception, and thus point-light displays may not be the ideal stimuli for action perception-specific research (Zucchini et al., 2023). Studies using point-light displays should recognize that the process of structure-from-motion interpretation required by point-light stimuli is not a part of real-life processing. In this regard, recent technological developments have made possible the use of, for example, naturalistic videos for the investigation of real-life action understanding using brain-imaging methods (de Gelder et al., 2004; Grèzes et al., 2004).

More naturalistic, full-body stimuli not only reflect real-life processing more accurately, but they also contain more information than point-light displays. The key finding of point-light display research on action perception was that motion information was sufficient for action perception, crucially as long as the temporal relations of the point light tokens were undisturbed (Bertenthal and Pinto, 1994). However, with full-body stimuli, there is additional form information, and thus it may not be the case that motion and temporal information are necessary for action perception. Indeed, Atkinson et al. (2004) and de Gelder et al. (2004) showed that emotional actions could be accurately distinguished from static full-body stimuli.

Thus, it is important to also move away from point-light displays and consider the processes of action perception associated with these more naturalistic images. Point-light stimuli highlighted the importance of motion and temporal information, although discussion has focused largely on the motion information. This, however, conflates two separate concepts, as motion information does not necessarily guarantee fluidity or that the movements follow the appropriate temporal order. Computational models (Giese and Poggio, 2003), neuronal data from monkeys (Russ et al., 2023), and human neuroimaging data (Cerliani et al., 2022) have highlighted the importance of temporal information, separate from that of motion information. With dynamic, full-body stimuli, we are able to attempt to disentangle the individual contributions of motion and temporal information, as well as addressing the effect of form information on action perception.

The use of naturalistic images also poses new challenges. The classical experimental approach has consisted of selecting a small set of stimuli and selecting which ones to use in the experiment based on results from a pilot sample of viewers. Following such a piloting procedure the observed consensus among individual subjective recognition established the basis for generalizing to the population at large. If a high proportion of responders reported perceiving a forward movement of the arm and closing of the hand as grasping a cup, then this counted as the meaning of the movement or as the action being performed. The “incorrect” or alternative responses were written off as due either to noise in the stimuli, to less than perfect performance of the actors, or to the participants' failing attention or imperfect understanding.

Two developments in the last decade have modified the landscape. One is that large databases of videos of natural actions have become available (Monfort et al., 2020). The other is that new platforms have made it possible to extend validation of stimuli from small groups to cohorts of hundreds of participants. Studies on action perception no longer have to be limited to a select few, often instrumental, actions, but videos of hundreds of full body actions are available. Furthermore, these actions can take place in a variety of real-world contexts (Dima et al., 2022; McMahon et al., 2023). Consequently, such action databases present a very large and diverse semantic action space with many more dimensions of variability than envisaged in classical studies. This situation increases the chances of observing individual variability in how one or another actor and action is perceived. Already Dittrich (1993) found that from point-light displays, locomotory actions were identified much more reliably than instrumental or communicative actions, and for some actions recognition accuracy was below 50%. As the use of naturalistic stimuli is relatively new, there are no data yet on the issue of variation in action recognition within different action categories.

The goal of this study is to investigate some of the issues associated with the use of naturalistic stimuli for studying action perception. Firstly, how do form, temporal, and motion information affect the recognition of actions in full-body, naturalistic stimuli? To do this, we presented action stimuli in three conditions: as still images, as dynamic videos, and as dynamic videos with the frame order scrambled to disrupt temporal continuity. To focus on the effects of body perception only, we blurred the faces of the actors and avoided any context effects by excluding tools and scenery (Wurm et al., 2012; Wurm and Schubotz, 2017). We predicted that normal dynamic videos would lead to the highest recognition accuracies, while the still images would lead to the lowest (Atkinson et al., 2004). Secondly, what can we learn about action perception from studying the errors underlying incorrect action attribution? To investigate this, we presented ten different social, instrumental, emotional, and communicative actions and participants were prompted to identify the action they perceived. The paradigm used was either a forced choice task, where they selected their response from a list of ten options, or a free description task, where they were free to describe the action in their own words. The free description task was included to gather an assessment of action understanding unbiased by the experiment labels and thereby to reflect participants' sampling of the semantic action space more accurately.

## 2 Methods

### 2.1 Participants

One hundred and nine undergraduate students (mean age = 21.1; age range = 18–34; 24 male, 83 female, 2 other) were recruited through the online participant recruitment system at Maastricht University. All participants had normal or corrected-to-normal vision and no history of psychiatric or neurological disorders. 88% of participants identified themselves as right-handed. All participants were fully informed about the experiment and provided written consent before commencing. As remuneration, participants received credit points. The experiment was approved by the Ethics Review Committee Psychology and Neuroscience (ERCPN) at Maastricht University and was conducted in accordance with the Declaration of Helsinki.

### 2.2 Stimuli

The stimuli consisted of six actors (all male) miming ten different actions (self-protecting [A1], greeting a friend [A2], expressing frustration [A3], telling off [A4], admitting a mistake [A5], brushing off [A6], peeling a banana [A7], picking berries [A8], searching for an object [A9], catching a ball [A10]). The first five actions (A1-A5) were classified as social/emotional, while the second half (A6-A10) consisted of instrumental actions. The actions were chosen to align with the putative action classes proposed by Orban et al. (2021). Action A1 is “defensive”, actions A2-A5 are “interpersonal”, action A6 is “self-directed”, actions A7 and A10 are “manipulation”, action A8 is “ingestion”, and action A9 is “reach”. The instructions given to actors to perform these actions and examples from each action category can be found in Supplementary Table S1.

Each actor, wearing uniform black clothing, was individually filmed performing five consecutive performances of each action. These segments were then cut into individual videos of each action performance, creating one-second long videos of 50 frames/s. In order to investigate the influence of temporal information (and its disruption) we decided to keep the duration of the videos fixed, so as to not include a confound in the results. All of the actions were fully completed within the one second. The background of each video was removed and replaced with a homogeneous green color (RGB = [98, 218, 149]) using the methodology developed by Lin et al. (2020). The actors' faces were blurred by estimating the face position using the Viola-Jones algorithm (Viola and Jones, 2001), setting a square around the estimated face position, and tracking the midpoint throughout the video using the Kanade-Lucas-Tomasi algorithm (Lucas and Kanade, 1981; Tomasi and Kanade, 1993). Finally, the midpoint served as the center of a Gaussian filter (size = [75, 75], sigma = 100) with the radius of the circle determined by the face detection algorithm. All the steps of the face blurring were completed using custom scripts in MATLAB.

In a previous validation study, 81 participants (mean age = 22; age range = 19–31; 59 female, 22 male) viewed all 300 of the resulting videos and categorized the action type in a forced-choice task. For each actor and action, the rendition with the highest recognition accuracy was then chosen to be used as a stimulus in this study. The average accuracy of the selected videos was 90.6% (range: 30–100%) with a chance-level accuracy of 10% (see Supplementary Table S2 for more information about the recognition accuracy per action). For the present study, all videos were converted to gray-scale (background RGB = [175, 175, 175]). To address our research questions, we also created two more stimuli from each video–a still image and a version of each video with the order of the frames time-scrambled. The procedures used to create these other stimulus conditions are described below.

#### 2.2.1 Still images

The frame-by-frame algorithm (Bockes and Vrabie, 2021) was employed to select the frame in each video that best represented the depicted action. For each video stimulus, an action from the pre-trained list of 339 actions was chosen as the ground truth. The algorithm then assigned each frame in the video a likelihood of that frame depicting the given action. The frame with the highest likelihood or “softmax” value was then chosen as the still stimulus (see Supplementary Figure S1 for an example). The frame-by-frame algorithm was run in Python, while the frame extraction was completed using custom scripts in MATLAB.

#### 2.2.2 Frame-scrambled videos

The frames of the original videos were also pseudo-randomized to create versions of the videos with the temporal order of the actions disrupted. The frames were reordered in blocks of 10 frames, with the constraint that the final block of the original video was placed into the middle and the frame-scrambled version did not begin with the same block of frames as the original. The constraints were chosen to ensure that the beginning and end of each frame-scrambled stimulus were different to the original, to increase the likelihood of action understanding disruption. Blocks of 10 frames were used, as using smaller subsets of frames resulted in significant visual flickering in the scrambled video. The scrambling procedure was completed using custom scripts in MATLAB.

With six actors performing ten actions and each combination represented in three different conditions (normal video, frame-scrambled video, still image), this resulted in a total of 180 unique stimuli.

### 2.3 Procedure

The study was completed on the online platform (Qualtrics, 2020). Each participant completed one of two tasks. The full list of 180 stimuli was split into two counterbalanced lists of 90 stimuli each. Participants were randomly allocated to one of two lists, resulting in four possible combinations of task and stimulus list. The stimulus lists were balanced to ensure that each contained an equal number of each action type, stimulus condition, and actor, with no repetition or overlap. Before completing the task, demographic and handedness data were collected from the participants.

#### 2.3.1 Forced choice task

In the forced choice task, participants saw each of the 90 stimuli in a random order, and after viewing each stimulus for one second, they were presented with a list of the ten possible action categories (Self-protecting, Greeting a friend, Expressing frustration, Telling off, Admitting a mistake, Brushing off, Peeling a banana, Picking berries, Searching for an object, Catching a ball). They had to select which action from the list they thought was being depicted in the viewed stimulus. The order of the items in the list randomly changed with each trial. The participants had an unlimited amount of time to choose their answer and could only select one option.

#### 2.3.2 Free description task

In the free description task, participants were also presented with each of the 90 stimuli in a random order with both the static and dynamic stimuli displayed for one second. After viewing each stimulus, the participants were instructed to describe the action in their own words, with a maximum of three words. A maximum of three words was set to encourage participants to describe the stimulus as directly as possible and ensure the experiment could be completed in a reasonable amount of time. Participants had unlimited time to respond before proceeding to the next stimulus.

In both tasks, participants were only able to watch each video once and they had an opportunity to take a break after viewing each tenth stimulus.

### 2.4 Analysis

#### 2.4.1 Forced choice task

Accuracies for each stimulus were calculated based on participants' responses. As the data were not normally distributed, the accuracies between the different stimulus conditions were assessed using generalized estimating equations (GEE) with the stimulus conditions (video, still, frame-scramble) and actions (10 levels) as fixed effects with Bonferroni corrections for multiple comparisons. GEE is a form of general linear model, which aims to take into account potential uncontrolled correlations between data points and thereby conduct a more robust analysis of repeated-measures data.

#### 2.4.2 Free description task

Participants' responses on the free description task were analyzed using two methods, one qualitative and one quantitative. The qualitative approach allowed us to consider the nuances of participants' responses and accurately incorporate them into our results, while the quantitative approach allowed for a more direct comparison with the quantitative results of the forced choice task. For a qualitative approach, framework analysis was used (Goldsmith, 2021). This is a form of thematic analysis, where the data are first explored to identify common themes within, and these themes are then used to index the data and map patterns.

The responses were also analyzed quantitatively using the Word2vec algorithm (Mikolov et al., 2013). This embedded each phrase response in a high-dimensional space based on its semantic similarity to pretrained data. Thus, each response received a vector indicating its phrasal embedding. In order to then find responses which were embedded near each other in the latent space, we used hierarchical density-based spatial clustering of applications with noise (HDBSCAN). This is a form of hierarchical clustering, which grouped the responses based on their embedding vectors and then identified clusters based on a minimum number of samples in a cluster (McInnes et al., 2017). We selected a minimum number of samples in a cluster of 5, which was intended to be small enough to capture as many clusters as possible in the wide semantic space available. Additionally, the response embeddings for all of the actions were also compared to each other using Representational Similarity Analysis (RSA) to investigate how similar responses were to each other both within action categories and between action categories (Kriegeskorte et al., 2008). Cosine similarity was used as the metric for the analysis.

## 3 Results

### 3.1 Forced choice task

**Figure 1:**
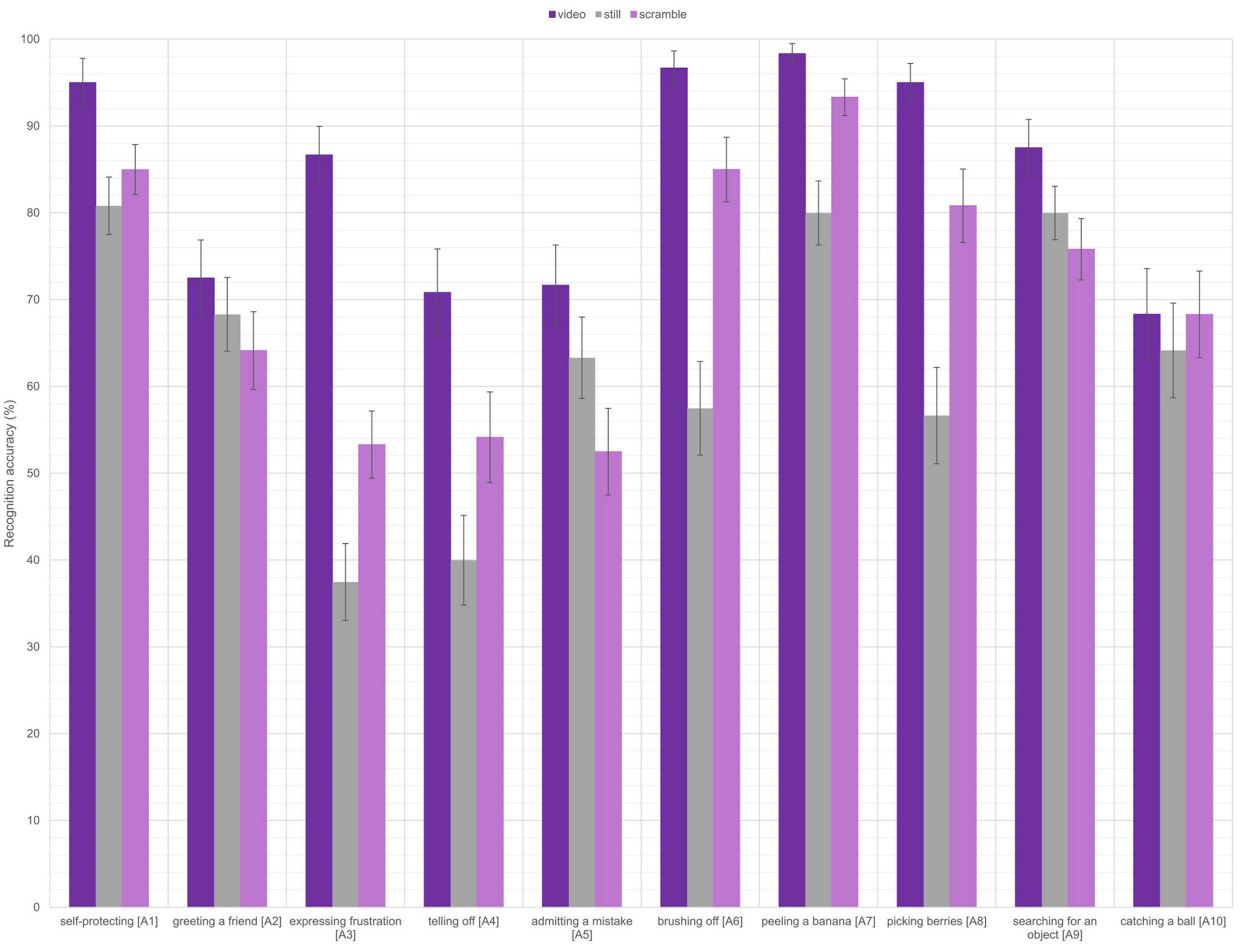
The recognition accuracies per condition on the forced choice task for each of the ten actions. Error bars represent the standard error.

For the forced choice task, the analysis showed significant main effects of condition [Wald χ^2^(2) = 150.796, *p* < 0.001] and action [Wald χ^2^(9) = 243.766, *p* < 0.001], as well as a significant interaction [Wald χ^2^(18) = 578.171, *p* < 0.001; see Figure 1]. Follow-up Bonferroni-corrected pairwise comparisons showed that video stimuli (mean = 84.23%, SE = 1.60%) resulted in significantly higher accuracy than both scrambled stimuli (mean = 71.22%, SE = 1.53%, *p* < 0.001) and still stimuli (mean = 62.80%, SE = 1.52%, *p* < 0.001). Scrambled stimuli also led to a significantly higher accuracy than the still stimuli (*p* < 0.001). There were also many significant pairwise comparisons between individual action categories. The clearest trends were that the “self-protecting” (mean = 86.94%, SE = 2.04%) and the “peeling a banana” (mean = 90.56%, SE = 1.78%) actions showed significantly higher accuracies (all *p* < 0.034) than seven of the other actions (all except the “searching for an object” action). Also, the “expressing frustration” (mean 207 = 59.17%, SE 2.50%), “telling off” (mean = 55.00%, SE = 3.71%), and “admitting a mistake” (mean = 62.50%, SE = 3.42%) actions showed significantly lower accuracies (all *p* < 0.006) than five of the other actions (all except “greeting a friend” and “catching a ball”).

Investigating the significant interaction and comparing the stimulus conditions per each action category revealed several different patterns of activity. For the “self-protecting”, “greeting a friend”, “admitting a mistake”, “searching for an object”, and “catching a ball” actions, there were no significant differences in accuracies between the stimulus conditions (normal videos, scrambled videos, still images). For the “expressing frustration” action, the normal video condition led to significantly higher accuracies for the videos than for the still images or the scrambled videos (both *p* < 0.001). There was no significant difference between the still images and scrambled videos. For the “telling off”, “peeling a banana”, and “picking berries” actions, normal videos led to a significantly higher accuracy than the still images (all *p* < 0.002). There were no differences between the scrambled videos and the normal videos or the still images. Finally, for the “brushing off” action, there was a significant difference between all three stimulus conditions. Videos led to significantly higher accuracies than scrambled videos (*p* = 0.048) and still images (*p* < 0.001), and scrambled videos also led to significantly higher accuracies than still images (*p* < 0.001).

**Figure 2:**
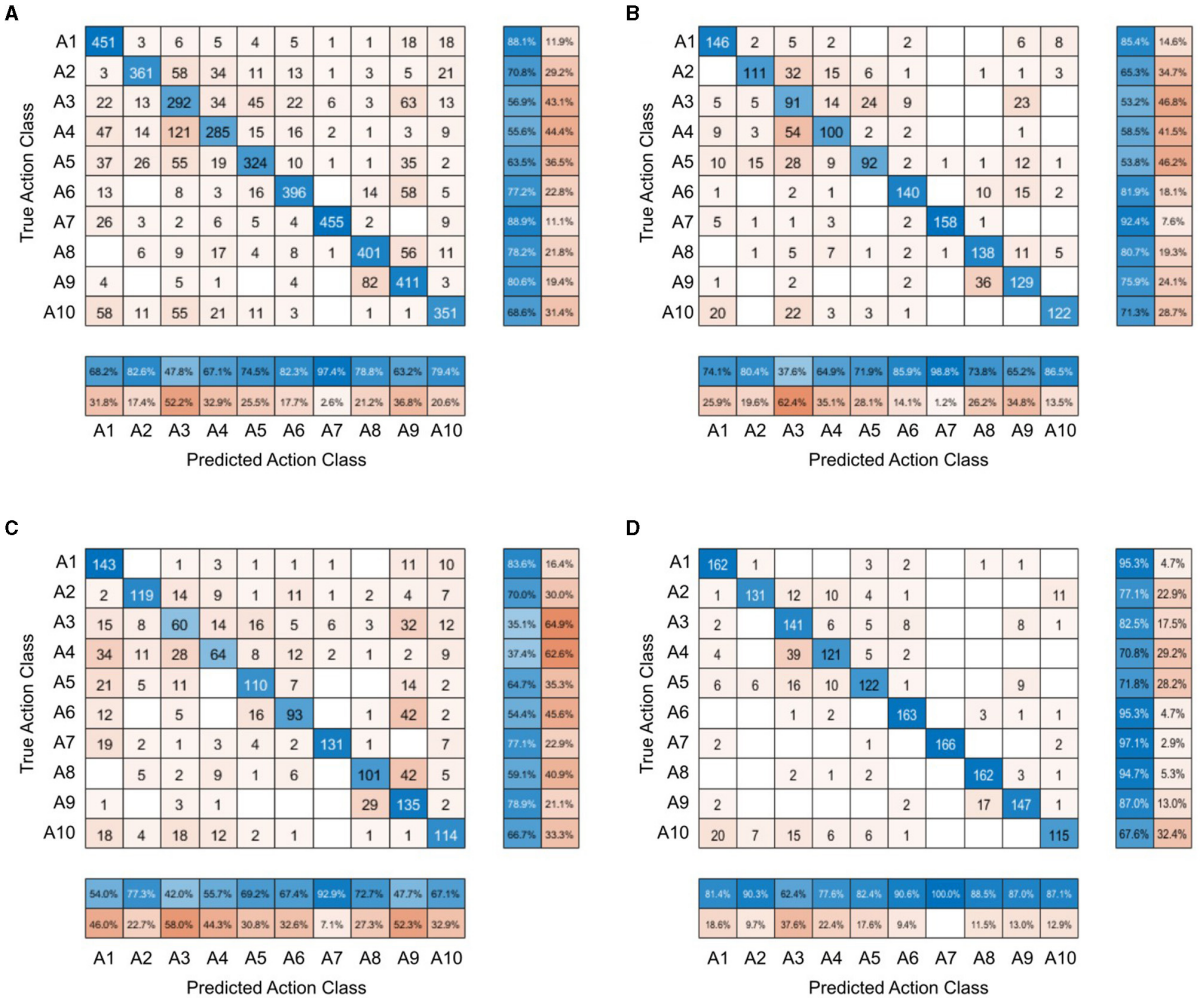
Confusion matrices of participants' responses on the forced choice task. Each row represents participants' responses to one action category. **(A)** Confusion matrix for all stimulus conditions combined. **(B)** Confusion matrix for the frame-scrambled video condition. **(C)** Confusion matrix for the still image condition. **(D)** Confusion matrix for the normal video condition.

A confusion matrix was created to show the distribution of participants' responses when categorizing the action depicted in the stimuli, across all stimulus conditions and also per each stimulus condition (see Figure 2). The matrix in Figure 2A shows that “greeting a friend”, “telling off”, “admitting a mistake”, and “catching a ball” were actions incorrectly identified as “expressing frustration” in more than 10% of trials. Conversely, in almost 12% of trials, “expressing frustration” was incorrectly labeled as “searching for an object”. The confusion matrices for each separate stimulus condition highlight a wider sampling of the semantic space increasing from the normal videos through the frame-scrambled videos to the still images. For the normal videos (Figure 2D), only 60% of the possible 100 true action class-predicted action class pairings occurred in the participants' responses, while for the scrambled videos (Figure 2B), 72% of them occurred and for the still images (Figure 2C), 84% of the pairings were present in the responses.

In order to compare our findings to previous research by Dittrich (1993) we divided our ten actions into emotional ones (actions A1–A5) and instrumental ones (actions A6–A10). We then compared the average recognition accuracies between these two action types using a Mann-Whitney *U* test. The test showed that instrumental actions resulted in a significantly higher recognition accuracy than emotional actions, *U* = 5374.000, *p* < 0.001.

### 3.2 Free description task

For the free description task, the responses were first assessed in terms of the number of words used. Participants were instructed to respond with a maximum of three words. On average, participants replied with 2.22 words, and this pattern remained consistent across all action categories (see Supplementary Figure S2).

**Figure 3:**
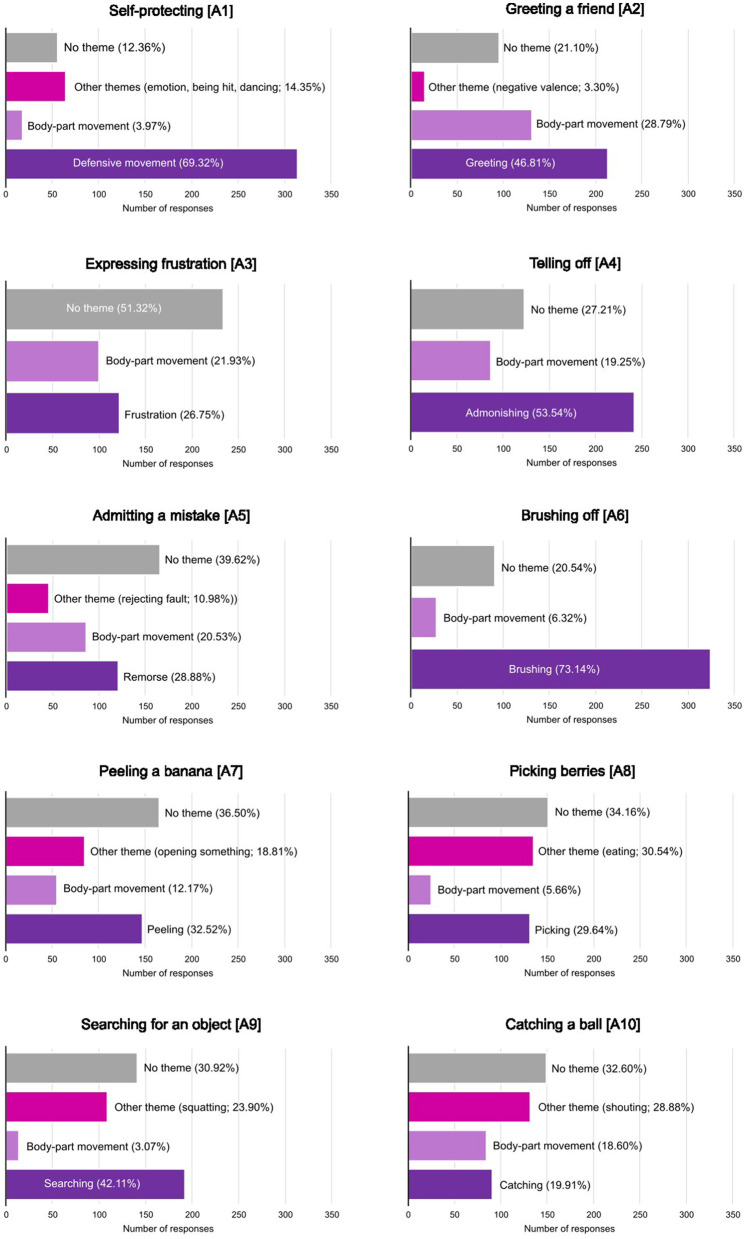
The distributions of responses on the free description task between the themes identified using framework analysis for each action. Percentages represent the proportion of the responses attributed to a given theme. Details of the themes can be found in Supplementary Table S1.

The framework analysis identified themes, which brought together the various responses for each of the action categories (see Figure 3). The list of all themes identified for each action can be found in Supplementary Table S3.

With a minimum cluster size of 5, the spatial clustering of semantic embedding analysis identified roughly 15 clusters for each action, however between 44.10 and 85.47% of responses were assigned to no cluster due to low similarity with other responses (see Table 1).

**Table 1:** The clusters of free description responses identified by clustering analysis of the Word2vec algorithm phrasal embeddings.

**Self-protecting:** Evading, dancing, defending, dodging, fighting, ducking, dodge, avoiding a slap, stepping aside, dodging a ball, avoiding, avoiding a punch, dodging an object, being scared/angry/shocked, leaning back, getting hit, avoiding something, dodging something, ducking away (50.22% uncategorized)
**Greeting a friend:** Hugging, hugging, hug, welcoming someone, greeting someone, high five, ready for hug, giving a hug, raising hands, hands up, raising arms, arms up, arms open, arms wide open, opening arms (58.39% uncategorized)
**Expressing frustration:** Frustration, standing, being upset/frustrated, being angry, being annoyed, shaking hands, looking for something, looking around (85.47% uncategorized)
**Telling off:** Arguing, gesturing no, Gesturing no, denying something, arms crossed, stopping something, crossing hands, crossing arms, being angry/frustrated, saying no, telling off, saying categorical no, saying stop, Saying no (64.05% uncategorized)
**Admitting a mistake:** Apologizing, apologizing, waiting, feeling ashamed, being sad, being shy, looking for something, looking down, hands up/behind back, standing (73.74% uncategorized)
**Brushing off:** Rubbing thigh, wiping dirt, rubbing pants, brushing leg, grabbing in pocket, removing dirt, looking for something, looking down, wiping leg, cleaning pants, Cleaning pants, cleaning the leg, cleans leg, wipe something away, Dusting off pants, shaking off, dusting off pants, brushing off trousers, to brush off, brushing dirt off, brushing off, brushing something off (44.10% uncategorized)
**Peeling a banana:** Fishing, peeling motion, opening a bottle, Peeling a banana, peeling a banana, peel a banana, eating, pouring something, opening a can, explaining something, opening something, open something, holding something, playing with hands (44.98% uncategorized)
**Picking berries:** Eating, searching, tasting, eating, tasting something, Eating something, picking berry, picking and eating, eating something, grabbing something, looking for something, picking something up, picking up food (49.35% uncategorized)
**Searching for an object:** Crouching, kneeling, petting a dog, Petting a dog, searching, bending over, hunched over, crouching down, picking something up, searching something, searching the floor, Searching for something, Searching/looking on ground, Looking for something, looking for something, searching for something (50.87% uncategorized)
**Catching a ball:** Shouting, screaming, begging, Catching a ball, catching a ball, ready to fight, being annoyed/frustrated, catching something, being scared, step/jump back, holding hands up (58.77% uncategorized)

**Figure 4:**
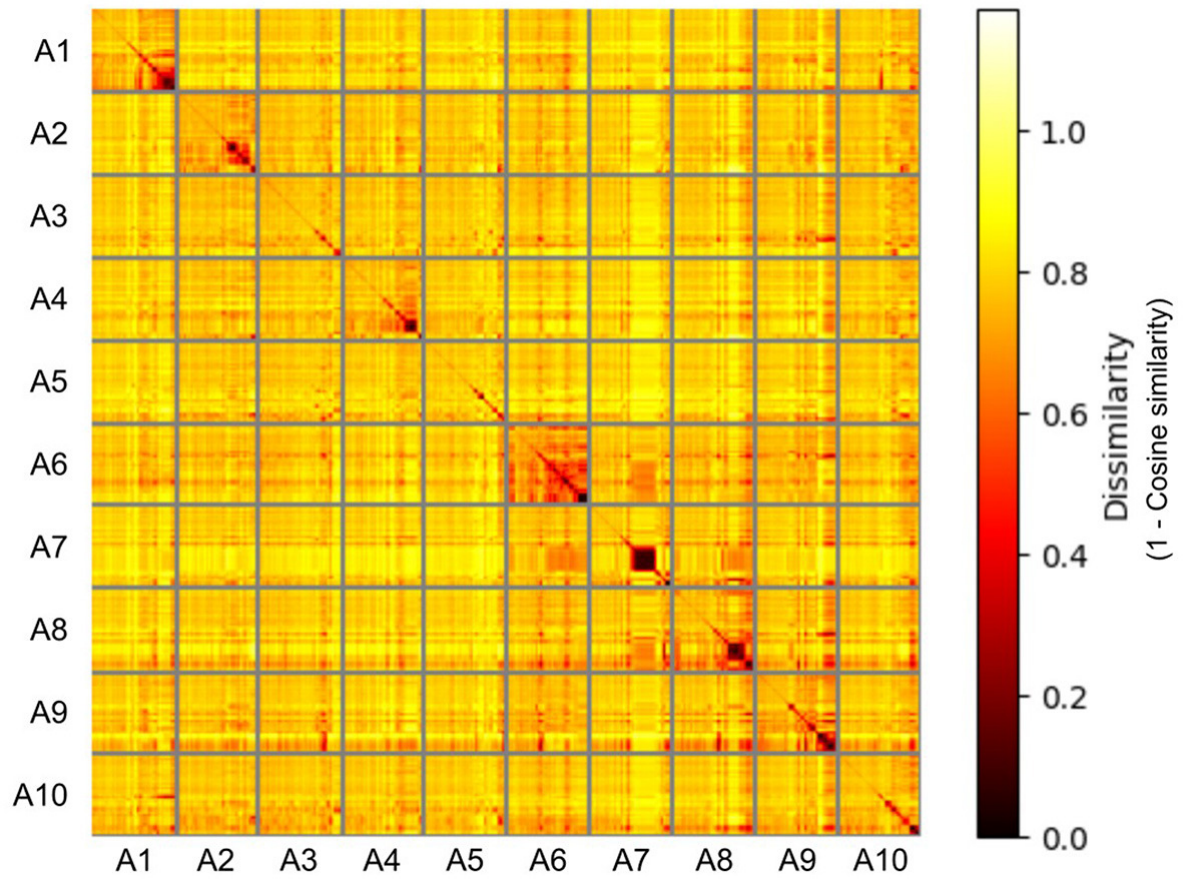
The representational dissimilarity matrix of the semantic response embeddings for the 10 different actions, with Cosine similarity used as the dissimilarity measure. Based on the responses from the free description task analyzed using the Word2vec model. (A1: self-protecting, A2: greeting a friend, A3: expressing frustration, A4: telling off, A5: admitting a mistake, A6: brushing off, A7: peeling a banana, A8: picking berries, A9: searching for an object, A10: catching a ball).

Representational Similarity Analysis was conducted on the response embeddings to visualize similarities between labels used for the different action categories (see Figure 4). Generally, there was a high dissimilarity between the responses used for different action categories, with almost all sources of similarity being from within-action responses. There was generally higher similarity between the responses for the instrumental actions (actions A6–A10). The responses for the “self-protecting” action showed particularly high dissimilarity from the responses for the other actions.

What emerged both from the framework analysis and the semantic embedding analysis, is that participants often resorted to describing or naming body parts and their movements rather than the actions themselves. To further investigate this, for each stimulus the responses that contributed to the “body movement” theme were counted, resulting in a proportion of body-part responses for each stimulus, ranging from 0 (no responses belonging to the “body movement” theme) to 0.44 (44% of all responses belonging to the “body movement” theme). This body-part proportion was then compared to the average accuracy for each stimulus. The body-part proportion showed a significant negative correlation with average accuracy [Spearman's ρ(180) = −0.392, *p* < 0.001], where stimuli with low accuracy showed a higher proportion of body-part movement responses. Split by the stimuli conditions, for videos this negative correlation was even greater [Spearman's ρ(60) = −0.583, *p* < 0.001], still present for scrambled videos [Spearmans' ρ(60) = −0.279, *p* =0.031], but not significant for still images [Spearman's ρ(60) = −0.200, *p* = 0.125].

## 4 Discussion

The aims of this study were to investigate how form, motion, and temporal information interact in the recognition of actions from full-light, whole-body stimuli, as well as to investigate individual differences and variability associated with action understanding. Participants viewed stimuli of full-body, single actor actions and had to complete one of two tasks–either they had to decide, which action from a list of 10 options was being performed in the stimulus (forced choice task), or they were free to describe the performed action in their own words (free description task). The stimuli were in one of three conditions, each lasting 1 second: a video of the action, a video with the order of the video frames scrambled to disrupt the temporal order, or a single, static representative frame from each video.

As we predicted, for the forced choice task, both excluding motion information and disrupting temporal information led to a decrease in recognition accuracy. Specifically, still images led to lower accuracies than scrambled videos, and both led to significantly lower accuracies than the intact videos. However, there were also specific actions, where this did not apply and there were no differences in accuracy levels between the three stimulus conditions (“greeting a friend”, “catching a ball”). For other action categories, the stimulus conditions had pronounced effects on the accuracy levels (“expressing frustration”, “picking berries”). Additionally, the free description task revealed two main types of error in semantic action understanding, misattribution of the incorrect action label and a focus on specific body part kinematics in the absence of action understanding.

### 4.1 Form, motion, and temporal information

Whereas studies using point-light displays suggested that motion information was sufficient for action recognition (Dittrich, 1993; Ziccarelli et al., 2022), we show that form information can also be sufficient for action recognition and adding motion and temporal information can, in some instances, further enhance accuracy. This aligns well with the proposal by Gärdenfors and Warglien (2012), who suggested that actions could be represented in an “action space” of forces acting upon a conceptual space. In this instance, the forces consist of the motion and temporal information, while the conceptual space represents the form information. Thus, unlike in point-light studies, which suggest that the action space requires only one dimension, we show that indeed action recognition can occupy the entire proposed two-dimensional space. This also aligns with the model of Giese and Poggio (2003), a hierarchical neural model for the perception of biological movement. Their model consists of form and motion pathways, which combine to allow action recognition. Both theoretical accounts emphasize the importance of motion and form information, which our data supports, as intact videos, containing motion and form information, led to the highest levels of action understanding. Additionally, both pathways of the Giese and Poggio (2003) model rely on information collected over multiple time-points and in the appropriate order, which our results also confirm to be important, as the videos with temporally scrambled frames resulted in lower action understanding.

However, while this trend generally holds, there were actions where form information was sufficient for achieving a high level of action understanding and adding motion and/or temporal information did not significantly raise the recognition accuracy. Specifically, the actions “self-protecting”, “greeting a friend”, “admitting a mistake”, “searching for an object”, and “catching a ball” resulted in similar accuracies for all three stimuli conditions. Seemingly for these actions, even in the instances of dynamic videos, participants relied solely on form information from a single temporal reference point to correctly identify the action. The “expressing frustration” action showed the highest recognition accuracy for the normal video condition with no differences between the still images and the scrambled videos. The confusion matrices show that in the scrambled video and still image conditions this action was frequently misidentified, particularly as the “searching for an object” action. The results show that this action in particular was reliant on the temporal information to clarify this confusion. The “telling off”, “peeling a banana”, and “picking berries” actions resulted in recognition accuracies, which were significantly higher for the normal videos than for the still images. The “brushing off” action showed significant increasingly higher accuracies for the still image, scrambled video, and then normal video conditions. All four of these actions thus suggest a pattern, where both the addition of the dynamic and temporal information enhanced action recognition.

### 4.2 Subjective variability in action understanding

Our findings also contribute to the investigation of errors in semantic action understanding. Firstly, we found that some actions led to higher recognition accuracy than others in the forced choice task. Our action stimuli can be divided into an emotional/social half and an instrumental half. Overall, accuracies were lower for the emotional actions compared to the instrumental actions, which contrasts with the results of Dittrich (1993), who found that locomotory actions were recognized more than social actions, while instrumental actions had the lowest recognition rates. The likely explanation for this is the presence of form information in our stimuli, whereas Dittrich (1993) used stimuli with only motion information. Instrumental actions are not only about movements of the human body, but about interaction with the physical environment, and so it follows that they would be more affected by the absence of form information than social actions, which may not be so reliant on interaction with surroundings (Hsiung et al., 2019).

There are also exceptions to this general trend–the “self-protecting” action, whilst being a social action, resulted in significantly higher recognition accuracy than all other actions except “peeling a banana” and “searching for an object”. This is likely due to the action's communication of threat, which has been found to be preferentially processed by the brain (Bannerman et al., 2009). The RSA of the free description task responses also found that the responses for the “self-protecting” action showed a high degree of dissimilarity from the responses for the other actions, further arguing that this action generally did not suffer from semantic confusion with other actions. On the other hand, the RSA also suggested a generally lower degree of dissimilarity between the responses of the instrumental actions. This suggests that for some participants the information provided in the instrumental action stimuli was sufficient for constraining their search of the semantic space to the instrumental actions, but not enough to help them distinguish between the individual actions.

The inclusion of the free description task and the analysis of its results allowed us to explore potential reasons for why some actions were less well understood than others, and where action understanding failed. The results showed two primary forms of error. Participants would either misinterpret the stimulus and identify it with an alternative semantic label or they would fail to reach a semantic interpretation of the stimulus and focus on the movement of individual body parts. The RSA of the response embeddings and the forced choice task confusion matrices showed that some of the low accuracy was due to action categories being mistaken for each other. The “self-protecting” and “catching a ball” actions and the “picking berries” and “searching for an object” actions were both mistaken for each other in the forced choice task and the word embeddings showed that there was some overlap in verbal descriptions of the actions in the free description task. In this case, both action pairs have similar kinematics, with the “self-protecting” and “catching a ball” actions both being performed with a full-body shift to the right, and both the “picking berries” and “searching for an object” actions consisting of a squatting or lowering of the whole body. Additionally, in the forced choice task, the “telling off” action was often incorrectly labeled as “expressing frustration”. The error here most likely originates from similarities at the semantic level, as admonishing someone most likely involves expressing frustration. Even when unbiased by the label options offered by the forced choice task, participants applied incorrect action labels to many of the stimuli. For instance, almost 19% of the free description responses for the “peeling a banana” action fit in the “opening an object” theme. This is again likely driven by the similarity in kinematics and the lack of additional context in the form of the objects being interacted with.

The confusion matrices of the forced choice task also highlight this wider sampling of the semantic space in the event of lower action recognition. In the forced choice task, there were ten different actions presented and ten different response options for each stimulus, resulting in one hundred possible true action-class and predicted action-class combinations. For the normal videos, only 60% of these possible pairings occurred in participants' responses, while for the scrambled videos 72% of them occurred and for the still images, 84% of the pairings were present in responses. In combination with the findings that the still images led to the worst overall action recognition, followed by the scrambled videos, and the normal videos resulting in the highest accuracy, this further shows the larger sampling of the available semantic space in the event of greater uncertainty.

In instances where participants were unable to recognize any action, they tended to instead describe the movements of individual body parts, often the hands (Wallbott, 1998; Poyo Solanas et al., 2020) or arms (Pollick et al., 2002; Sawada et al., 2003). Interestingly, this was the case for dynamic stimuli only. de Gelder and Poyo Solanas (2021) suggested that behaviorally relevant information from body expressions, such as emotional category, is coded at the mid-feature level, which occupies the space between low-level visual features and high-level semantic concepts. Examples of such features include “limb contraction” and “head orientation”. This aligns well with our results, as when participants lacked action understanding, they tended to concentrate on midlevel features when describing the actions presented in dynamic stimuli. The focus on the hands in particular in participants' responses also matches previous findings on emotions being particularly well perceived from the movements of hands (Blythe et al., 2023).

It is also worth noting that in our quantitative analysis of the free description task results, we found that for many of the actions, a large proportion of the responses did not fit well into any of the identified semantic clusters. This further highlights the large size of the available semantic space when naturalistic action stimuli are utilized, and not only is the semantic space large, but its breadth is also utilized by participants. Future studies utilizing large naturalistic datasets may wish to take into account this wide-ranging sampling of the semantic space, on top of existing methodologies of rating similarities between actions on a variety of physical and semantic features (Dima et al., 2022; McMahon et al., 2023).

### 4.3 Neural basis of visual action perception

Combining all of our results suggests that utilizing naturalistic stimuli for investigating the neural basis of visual action perception and for computational modeling needs to take into account many factors involved in achieving accurate action perception. We find that there are actions for which motion information is sufficient for accurate recognition (as shown by older point-light studies), other actions for which form information is sufficient, and yet other actions where the combination of form and motion information leads to the highest accuracy. Additionally, temporal information is highly important for some actions and not necessary for the perception of others. Our findings indicate that hierarchical models of action perception may need to account for this varying reliance on form and motion information (Parisi et al., 2015). In the context of hierarchical models, our findings do suggest that kinematics of body-parts are a relevant step in the process of action recognition (Hamilton and Grafton, 2008; Boyer et al., 2017), but only when those kinematics are directly present, as in the case of dynamic stimuli. Aside from these issues related to the presentation format of the images, there are other factors at stake that pertain to the semantics of the images. Some actions with high behavioral relevance (threat-related) have a higher recognition accuracy than others, even other emotional actions and instrumental actions require additional contextual information to allow for a high-level of recognition (Wurm and Schubotz, 2012, 2017; Wurm et al., 2012). Finally, natural action recognition seems to consist of a search in the available semantic space (Vinton et al., 2024), which is guided and constrained by the available information, whether it is form, motion, temporal, or contextual.

### 4.4 Limitations

Our study contains a gender imbalance in two ways - our participant sample consisted of significantly more females than any other gender (76% of the whole sample), and all of the stimuli consisted of male actors performing the actions. Past research has shown that males showed greater activation in attention-related brain areas for images of other bodies, while females showed greater activation for images of their own bodies, suggesting a gender-difference in salience stimuli (Burke et al., 2019). Other work has found no significant gender-differences in understanding others' actions and intentions (Pavlova, 2009). The current study does not allow us to investigate this systematically, due to the single gender present in the stimuli and the significant imbalance in the participant sample, however, it is important to keep these considerations in mind in light of our findings. Although we investigated the perception of actions with naturalistic body movements, we did not include contextual (i.e., scene) influences, which may have made the stimulus set more ecologically valid (den Stock et al., 2014). However, not choosing to do so allowed us to control for those aspects and focus on motion, form, and temporal processing.

For the free description task, there are also two important considerations to keep in mind. Firstly, participants were limited to responding in a maximum of three words. This may have prevented participants from being able to fully describe their perceptions. However, we find that on average participants replied with 2.22 words, suggesting that participants were not limited in their responses. Additionally, we did not assess the English proficiency of our participants and so variability in English ability may have also played a role, which we have not accounted for.

### 4.5 Conclusion

Our results contribute to our understanding of the objective and subjective dimensions of naturalistic action understanding. We show that form, motion, and temporal information all seem to be important for the process of action recognition, as suggested by various psychological and neuroscientific models, but we also show that the contributions of these sources of information do not have to be equal, as there are instances where form information is sufficient for action understanding and nothing is gained by adding temporal and motion information. This complements studies with point-light stimuli, which show that for some examples, only motion information is required for action recognition. Additionally, we highlight the two main errors in semantic action understanding, incorrect semantic labeling and a focus on kinematic descriptions of body-part movements, and elaborate on how these contribute to our understanding of action perception.

## Data availability statement

The datasets presented in this study can be found in online repositories. The names of the repository/repositories and accession number(s) can be found below: https://osf.io/yj437.

## Ethics statement

The studies involving humans were approved by Ethics Review Committee Psychology and Neuroscience (ERCPN), Maastricht University. The studies were conducted in accordance with the local legislation and institutional requirements. The participants provided their written informed consent to participate in this study.

## Author contributions

VS: Conceptualization, Data curation, Formal analysis, Investigation, Methodology, Project administration, Software, Validation, Visualization, Writing – original draft, Writing – review & editing. MP: Conceptualization, Investigation, Methodology, Supervision, Writing – review & editing. EF: Methodology, Validation, Resources, Writing – review & editing. BG: Conceptualization, Funding acquisition, Methodology, Project administration, Supervision, Writing – review & editing.
