# Supplementary material for: Differential contributions of body form, motion, and temporal information to subjective action understanding in naturalistic stimuli
Source: Front Integr Neurosci. 2024 Mar 12;18:1302960. doi: 10.3389/fnint.2024.1302960 (PMC10963482; doi:10.3389/fnint.2024.1302960)
Supplement: Supplementary file 1 [file Data_Sheet_1.pdf]

Supplementary Material

1 SUPPLEMENTARY FIGURES AND TABLES

1.1 Figures

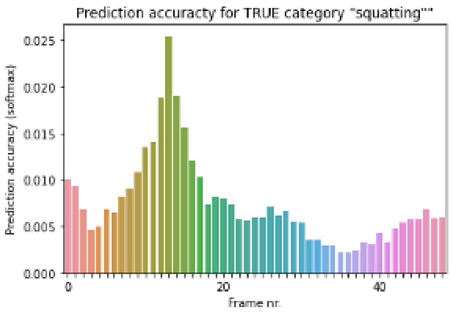

Figure S1. An example result of the frame-by-frame algorithm analysis for a video stimulus from the 'Searching for an object' category. Frame 15 was chosen as the static stimulus, as it had the highest softmax accuracy for the corresponding 'squatting' action, which was chosen as the ground truth for this action category (one of 339 actions available, which the frame-by-frame algorithm was pre-trained on).

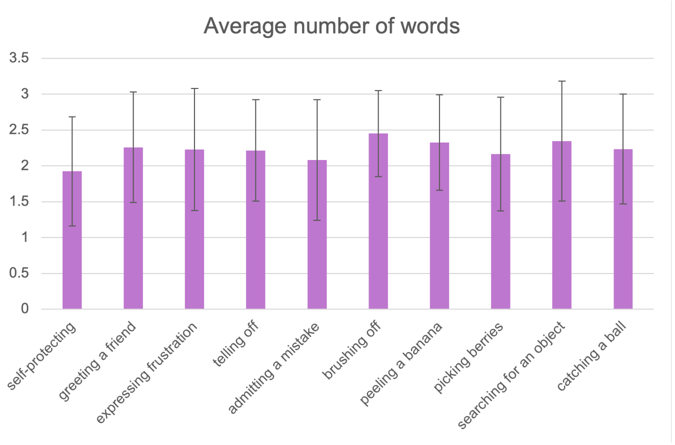

Figure S2. The average number of words used in the free description task responses for each category. Error bars represent the standard deviations.

1.2 Tables

Table S1: The instructions given to the actors for each of the action categories, along with an example still stimulus for each.

Action	Actor instructions	Example stimulus

Self-protecting [A1]	You are walking, when you see a stone flying in your direction, you are forced to react suddenly to dodge it.	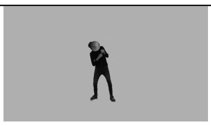
Greeting a friend [A2]	After the lockdown, you are free to walk in the street when you spot a good friend which you do not see since a lot, you start welcoming him/her since far away	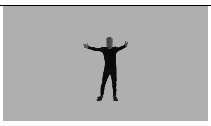
Expressing frustration [A3]	You feel your wallet is with you but then you realize you left it in a bar (react: you use your whole-body and bang your arms)	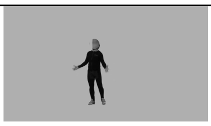
Telling off [A4]	There is a bunch of teenagers/kids fooling around, you approach to make them stop (dominant posture)	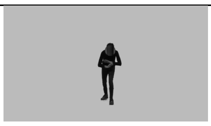
Admitting a mistake [A5]	Your boss tells you that you made a stupid mistake and you accept that s/he is right	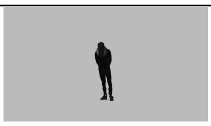
Brushing off [A6]	You are working in the kitchen, the doorbell is ringing, you quickly check yourself and you see that you have some flour on your pants, you brush it off. (5 movements, if right-handed they brush the left leg and vice versa)	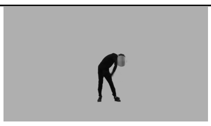
Peeling a banana [A7]	You forget to have lunch, you are then very hungry, someone just gave you a banana (peel it 3 times)	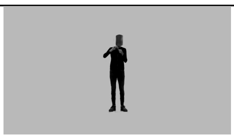

Picking berries [A8]	You are walking and you see a bush of blackberries: you bend over and pick 3 of them (one from your left, one from the center, and one from your right)	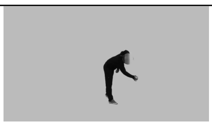
Searching for an object [A9]	You need a specific tool located on a very low shelf, you squat down and just look for it (no gesture), you found it and stand up	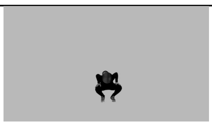
Catching a ball [A10]	Somebody thrown a ball at you, you see it approaching and you get ready to catch it (do not approach the ball, just get ready to catch it)	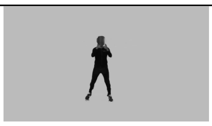

Action	Action Recognition Accuracy
Self-protecting [A1]	94.19%
Greeting a friend [A2]	87.00%
Expressing frustration [A3]	77.63%
Telling off [A4]	66.54%
Admitting a mistake [A5]	77.99%
Brushing off [A6]	100%
Peeling a banana [A7]	100%
Picking berries [A8]	98.15%
Searching for an object [A9]	98.49%
Catching a ball [A10]	75.46%

Table S2. The average accuracies for each of the action categories from the pilot validation study.

Table S3: The themes of the responses on the free description task for each action identified using framework analysis. The brackets contain example responses for each identified theme.

Action	Themes

Self-protecting [A1]	1. Defensive movement (pulling back, avoiding, ducking,...)<br>2. Body movement (shifting shoulder, body to side, stretching hand back,...)<br>3. Emotion (frightened, being shocked, being scared,...)<br>4. Being hit (getting hit, getting punched, fighting,...)<br>5. Dancing (dance move, dancing, dance smoothly,...)
Greeting a friend [A2]	1. Greeting (giving a hug, hugging, giving a high five, welcoming someone,...)<br>2. Body movement (swinging arms open, arms wide open, raising arms,...)<br>3. Negative valence (annoyed, disappointed, provoking someone,...)
Expressing frustration [A3]	1. Frustration (being annoyed, yelling, angry movement,...)<br>2. Body movement (swinging arms back, patting leg, arms down,...)
Telling off [A4]	1. Admonishing (argue, deny, motioning no, refusing,...)<br>2. Body movement (hands down, shaking arms, leaning forwards)
Admitting a mistake [A5]	1. Remorse (apologising, acting remorseful,...)<br>2. Body movement (swing arms, touching legs,...)<br>3. Rejecting fault (arguing, denying fault,...)<br>4. Shyness (standing shy, being shy, shy pose,...)
Brushing clean [A6]	1. Brushing (dust knee off, cleaning pants, wiping thigh,...)<br>2. Body movement (hit on knee, clap on thigh, head down,...)
Peeling a banana [A7]	1. Peeling (peel banana, peeling, unwrapping something,...)<br>2. Opening something (open can, opening bottle, opening a wrap,...)<br>3. Body movement (playing with hands, hand movements, moving hand down,...)

Picking berries [A8]	1. Picking (pick fruit, grabbing food, picking something, picking grapes,...)<br>2. Eating (eating something small, to eat, eating as snack, tasting,...)<br>3. Body movement (hand to mouth, putting in mouth, hands down, hand on knee,...)
Searching for an object [A9]	1. Searching (looking around, look for something, observe, watch closely,...)<br>2. Squatting (crouched and careful, to kneel, hunched over, ducking,...)<br>3. Body movement (moving his hand, patting your hand, grabbing foot,...)
Catching a ball [A10]	1. Catching (waiting for ball, catch ball,...)<br>2. Shouting (yelling, screaming, calling for something,...)<br>3. Body movement (to move hands, hands holding head, vertical arms raised,...)<br>4. Defence (defending oneself, evasion, cower,...)
